# Ultrafast computation of left ventricular ejection fraction using temporal intensity variation in cine steady-state free precession cardiac MR images with or without contrast

**DOI:** 10.1186/1532-429X-18-S1-O85

**Published:** 2016-01-27

**Authors:** Amol Pednekar, Debra Dees, Melissa Andrews, Benjamin Cheong, Raja Muthupillai

**Affiliations:** 1 Philips Healthcare, Houston, TX USA; 2 Radiology, CHI St. Luke's Health, Houston, TX USA

## Background

Adaptation of CMR in clinical setting among non-experts is limited due to the time-consuming and labor-intensive nature of both CMR data acquisition and post-processing. In many clinical instances, e.g., myocardial viability (MV), it is time efficient to acquire LV cine SSFP images after contrast administration. In this study we clinically validate an ultrafast algorithm that computes LVEF as an extension of the data acquisition by using temporal intensity variation (TIV) in cine SSFP images acquired with or without contrast.

## Methods

**Figure 1:**
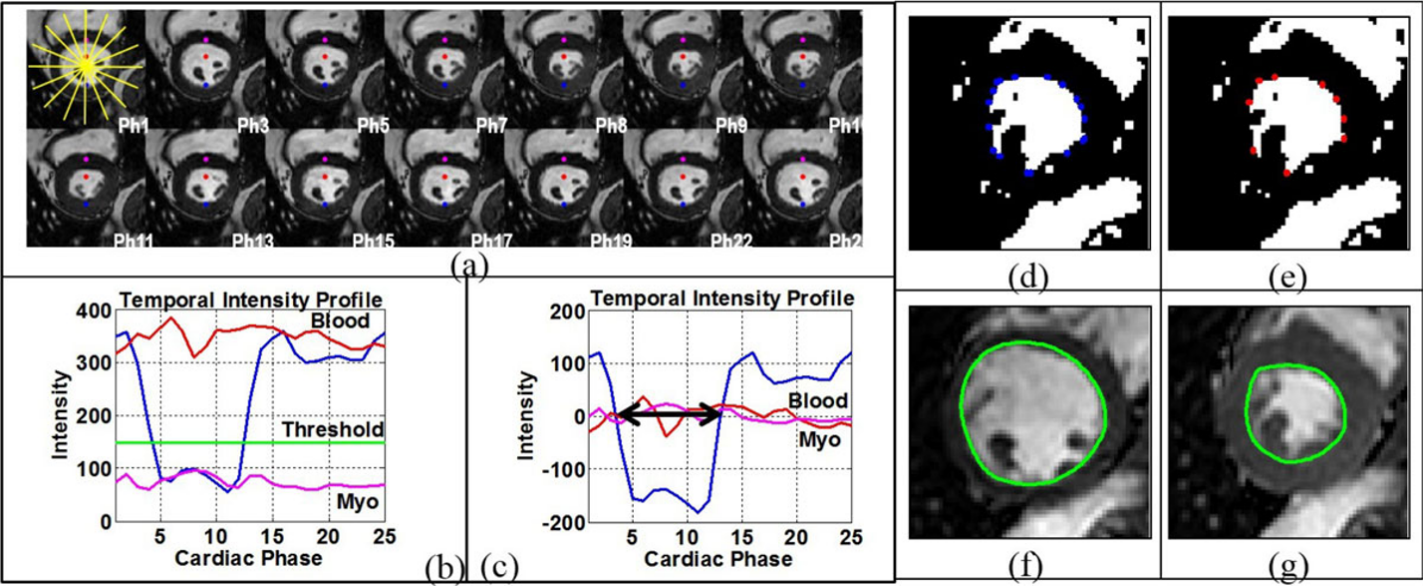
**Sample images illustrating the algorithmic steps for the automatic classification of partial-volume pixels and delineation of endocardial contour**. Time-intensity (TI) profiles, which show the variation in intensity over time, were created for individual pixels along 8 radial lines centered on the centroid of the identified left ventricular region (a). Panel c shows zero-clamped TI profiles of the same pixels depicted in b, with the mean signal intensity over the cardiac cycle subtracted from the signal intensity at each time point.The convex hull was fitted to the thresholded images (blue corners of the polygon) (d). The curvature and spatial proximity to adjacent corner criterion were used to select the salient points (red dots) of the convex hull (e). A piecewise closed Bezier curve of second order geometric continuity was fitted through the salient points of the convex hull. The corresponding endocardial contours are shown in green for the end-diastolic (f) and end-systolic (g) phases.

All imaging for this prospective, IRB approved study, was performed on a 1.5T commercial MR scanner (Achieva, Philips Healthcare). Study consisted of 16 volunteers (8 m/8 f; 38(27-54)yrs) and 44 patients (32 m/12 f; 52(17-83)yrs). In 7/28 MV patients, cine images were acquired within 10 min after administration of 0.2 mmol/kg of Gd-DTPA. CMR expert drawn myocardial contours served as the reference. The LVEF computation program written in MATLAB automatically runs in the background as an extension of the acquisition protocol on the scanner console (Intel Xeon, 3.20 GHz, 6 GB RAM) and does not affect the subsequent data acquisition. The key algorithmic steps are pictorially represented in Fig. 1 [1]. A Bland-Altman (BA) analysis was performed to quantitatively assess the effects of morphologic variation across the left ventricle on the accuracy of the algorithm.

## Results

The total computation time for LV stack was < 2.5 s. The algorithm successfully delineated the endocardial boundary with clinically acceptable accuracy in 1047 (93%) of the 1131 SA slices. The algorithm-derived contours for clinically unacceptable slices, which had the LV outflow tract and an extremely small (< 2 cc) LV section, were retraced manually. The bias and standard deviation of the difference for the apical, mid-cavity, and basal regions (Table 1) indicate that underestimation of the ESV primarily occurred in the mid-cavity region, which was due to the thickened dominant papillary muscles blending with the endocardium.

**Table 1:** Mean bias and standard deviation of the difference between the manually and automatically derived contours

	Apical EDV (mL, %)	Mid EDV (mL, %)	Basal EDV (mL, %)	Total EDV (mL, %)	Apical SV (mL, %)	Mid ESV (mL, %)	Basal ESV (mL, %)	Total ESV (mL, %)	EF (%)	EF# (%)
Bias	0.2, 0.0	-0.3, 0.0	-1.5, -0.4	-0.8, -0.4	1.5, 1.3	4.5, 6.0	-0.3, -0.2	5.6, 7.0	-3.6	-5.4
SDD	3.9, 1.6	8.0, 4.0	5.1, 2.8	14.2, 7.1	3.9, 3.8	7.8, 8.3	3.6, 4.1	11, 11.8	3.9	5.6

Bias = mean difference; EDV = end-diastolic volume; EF = ejection fraction; ESV = end-systolic volume; Mid = mid-cavity; SDD = standard deviation of the difference. Values computed by performing a Bland-Altman analysis. Results presented in mL and % error (ie, percentage of the total left ventricular volume calculated by manual delineation).

# EF computed by rejecting the clinically unacceptable contours (ie, the area was set to zero for these contours).

## Conclusions

Compared to previously reported in-line LV segmentation algorithms [2, 3], the computational cost of the proposed algorithm appears much lower (2.5 s for the entire stack versus 10-15 s per slice). The reliance on the temporal variation in signal intensity makes this approach attractive when evaluating LV volumes after contrast administration, which could improve work-flow in a clinical setting. The only user interaction required is to specify the basal and apical slices. Moreover, it does not put a constraint of acquiring the LV stack from base to apex. Temporal intensity variation based LVEF computation is clinically accurate and works across large range of LV shapes, wall motion, and even with post-contrast cine SSFP imaging. This computationally cost-effective algorithm allows CMR session to be more fluent by seamlessly integrating data acquisition and post-processing.
